# Adult-onset Primary Hemophagocytic Lymphohistiocytosis: Reporting a Rare Case with Review of Literature

**DOI:** 10.7759/cureus.6723

**Published:** 2020-01-21

**Authors:** Fatima Memon, Jawad Ahmed, Farheen Malik, Junaid Ahmad, Danish Ahmed Memon

**Affiliations:** 1 Internal Medicine, Dow University of Health Sciences, Karachi, PAK; 2 Pediatrics, Dow University of Health Sciences, Karachi, PAK; 3 Internal Medicine, Liaquat University of Medical and Health Sciences, Jamshoro, PAK; 4 Internal Medicine, Aga Khan University Hospital, Karachi, PAK

**Keywords:** hemophagocytic lymphohistiocytosis, adult-onset, hlh, autoimmune, t lymphocytes

## Abstract

Hemophagocytic lymphohistiocytosis (HLH) is an uncommon, aggressive hematological syndrome. It is caused by an increased and unchecked proliferation of T lymphocytes and histiocytes. These cells secrete a large number of inflammatory cytokines and infiltrate various tissues causing multi-organ system failure. HLH may be primary or associated with different types of infections, autoimmune disorders, or malignancies. Primary or familial HLH is fatal and is frequently considered a disorder of infants and young children. Only a few cases of primary HLH have been reported in adults. We present a case of a 37-year-old man who presented with fever, pancytopenia, and hepatosplenomegaly. Lymph node biopsy showed collections of histiocytes with lymphoplasmacytic cells. After excluding all the secondary causes a final diagnosis of primary HLH was made. The patient was started on HLH-2004 protocol (etoposide, cyclosporin A, dexamethasone) along with empiric antituberculous drugs as necrotic granulomas were also noted in the biopsy. HLH has a very poor prognosis and familiarity with clinical symptoms, and diagnostic criteria can aid in timely diagnosis.

## Introduction

Hemophagocytic lymphohistiocytosis (HLH) is a rare and fatal hematological disorder which results due to overactivation of the immune response. An inapt activation of T lymphocytes and histiocytes occurs, which causes these hyperactive immune cells to start attacking the blood cells, bone marrow, spleen, and other organs. Inflammatory cytokines are also produced in excess, creating a “cytokine storm” which results in clinical symptoms such as fever and rash [1]. The most common clinical manifestations of HLH are fever, hepatomegaly, and splenomegaly. HLH can either be primary or secondary. Primary or familial HLH occurs almost exclusively in the pediatric population, manifesting itself in the first two years of life [2]. Secondary HLH mostly arises in association with malignancies, autoimmune disorders or infections [3]. Primary HLH in adults is an extremely rare entity, with a few scattered cases reported in the literature. HLH has a high mortality rate along with its diagnostic challenge due to its various clinical presentations and aggressive treatment makes this disease an interesting one to report. Here we report a case of a 37-year-old man with primary HLH presenting with complaints of prolonged fever, pancytopenia, and hepatosplenomegaly.

## Case presentation

A 37-year-old man presented to the outpatient department of Agha Khan University Hospital with complaints of fever for five months and cough for one month. His fever was recorded up to 100.4°F, and physical examination revealed hepatosplenomegaly without any palpable lymph nodes. There was a history of undocumented weight loss.

Keeping an infectious cause in mind, he was started on empiric antibiotics (ceftriaxone 2 g and azithromycin 500 mg once daily) and baseline investigations were sent. Reports are summarized in Table 1.

**Table 1:** Summary of baseline investigations.

Parameters	Reports	Comments
Hemoglobin (Hb)	9.6 g/dL	Decreased
Red blood cell (RBC) count	4.4x10^6^/µL	Normal
Mean corpuscular volume (MCV)	72 fL	Decreased
Total leukocyte count (TLC)	1.0x10^3^/µL	Decreased
Neutrophils	74%	Normal
Platelets	64 x10^3^/µL	Decreased
Total bilirubin	0.6 mg/dL	Normal
Direct bilirubin	0.2 mg/dL	Normal
Indirect bilirubin	0.4 mg/dL	Normal
Gamma-glutamyl transferase (GGT)	130 IU/L	Raised
Alanine aminotransferase (ALT)	131 IU/L	Raised
Alkaline phosphatase (ALP)	199 IU/L	Raised
Aspartate aminotransferase (AST)	234 IU/L	Raised
Lactate dehydrogenase (LDH)	1051 IU/L	Raised
Ferritin	25903.5 ng/mL	Raised
Triglyceride	291 mg/dL	Raised

**Figure 1:**
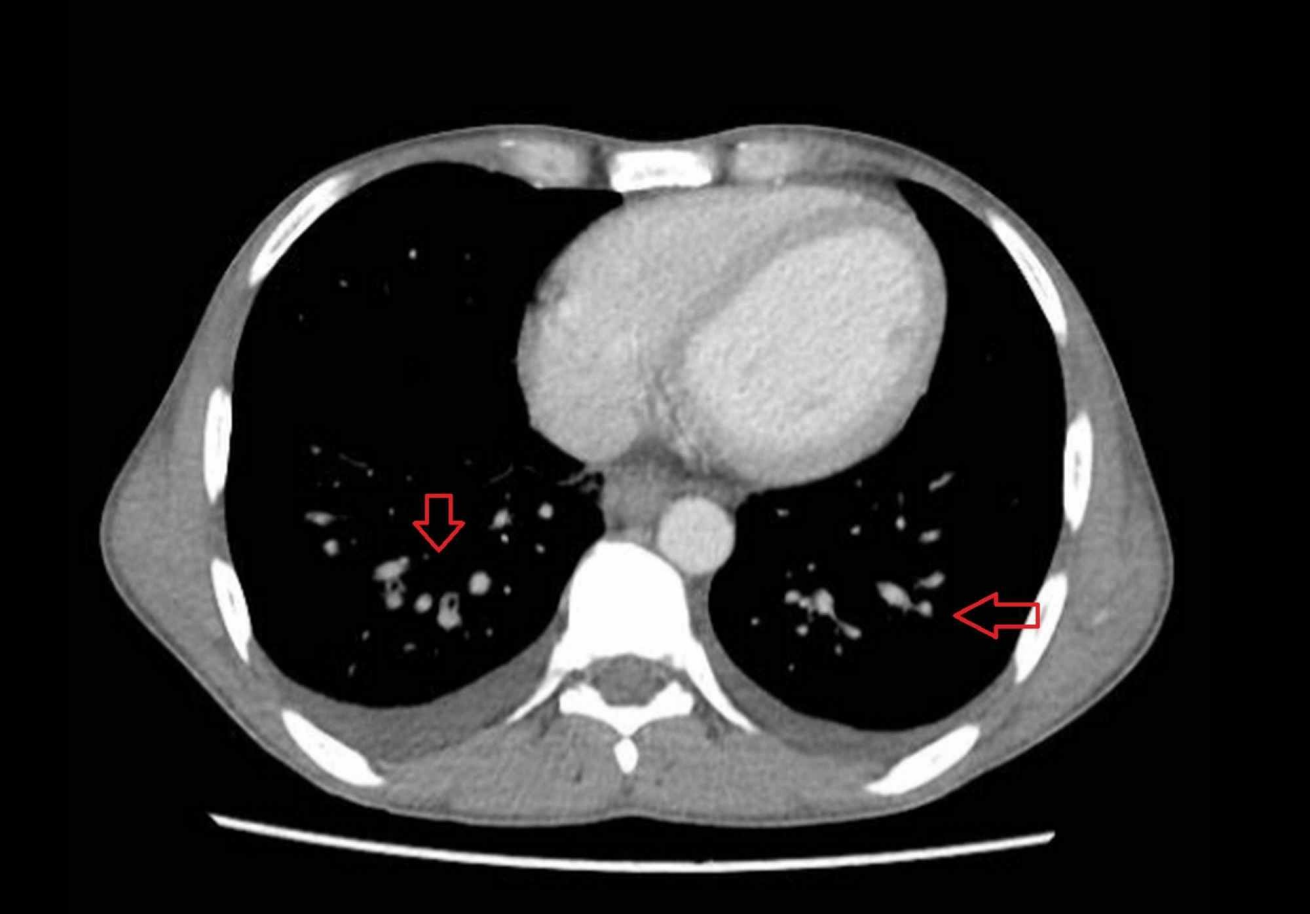
CT scan of chest showing variable-sized nodules (red arrows) in bilateral lung bases.

**Figure 2:**
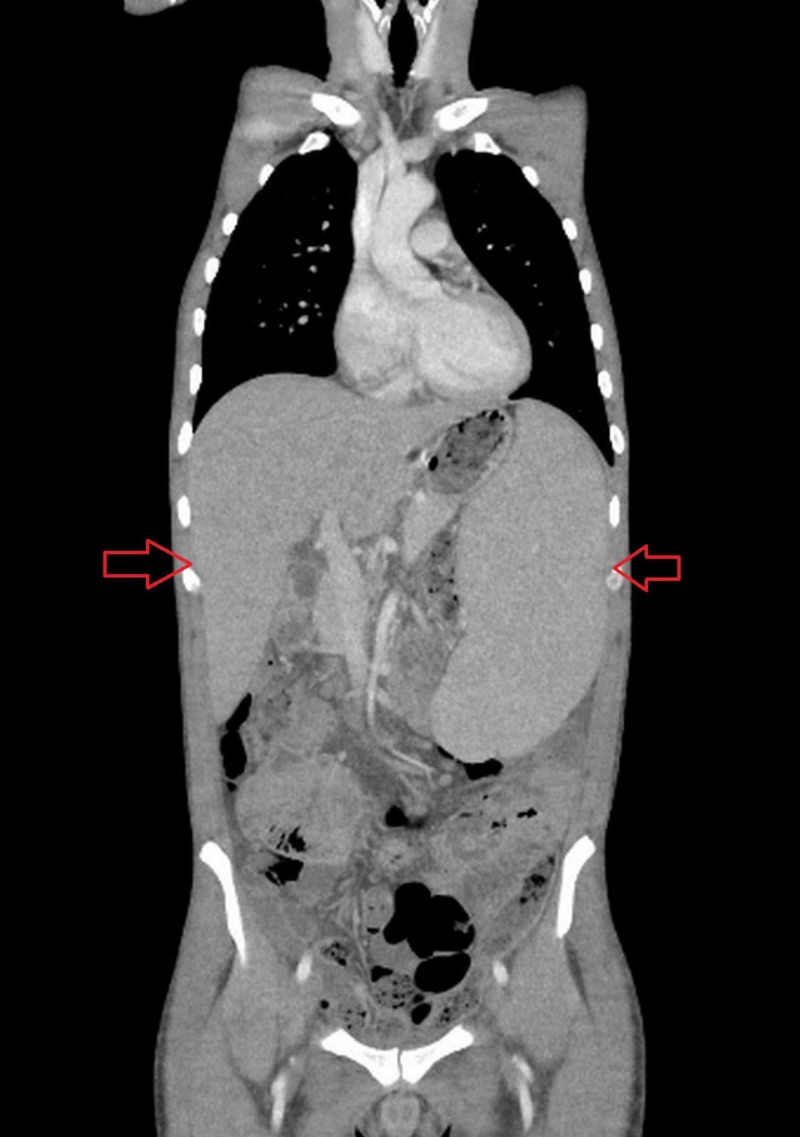
CT CAP showing hepatosplenomegaly (red arrows). CAP: chest, abdomen, and pelvis.

Due to hepatosplenomegaly and abnormal baseline investigations, he underwent computed tomography (CT) scan of chest, abdomen, and pelvis (CAP) to look for any malignancy or lymphadenopathy. CT CAP showed multiple, variable-sized nodules in bilateral lung bases (Figure 1) and coarse interlobular septal thickening. Mild bilateral pleural effusion was seen, but there was no evidence of pneumothorax, consolidation, or infiltrate. Retroperitoneal, para-aortic, and mediastinal lymph nodes were enlarged. Mild ascites and congestion of mesentery with bowel wall edema were also noted. Hepatosplenomegaly was seen as well (Figure 2).

Viral and infectious etiologies were ruled out. Epstein‐Barr virus (EBV), cytomegalovirus (CMV), hepatitis B and C virus (HBV and HCV), infectious mononucleosis, Legionella, malarial parasites, Mycoplasma pneumonia, Brucella, Mycobacterium tuberculosis, Nocardia, fungal smears, and HIV screening were all negative. Blood and urine cultures were also negative. An extensive autoimmune workup was done which was negative. Bone marrow biopsy and aspirate showed cellular marrow with trilineage hematopoiesis. No nonhematopoietic clump or heme parasite was identified on the stained smears. Bone trephine biopsy showed processing and hemorrhagic artifacts, intact architecture, and normal cellularity for age. Cellular areas showed the presence of erythroid and myeloid precursors. Megakaryocytes were also found. No granuloma or infiltration was identified in sections. On the basis of CT scan findings, the possibility of lymphoma or leukemia was raised and CT-guided abdominal lymph node biopsy was done. The patient was discharged in a stable condition and was advised to follow up with the biopsy report.

A week later, the patient arrived in a confused state with tachypnea in the emergency department. Repeated labs indicated pancytopenia with a decreasing hemoglobin of 7.8 g/dL, red blood cell count of 3.37x10^6^/µL, total leukocyte count of 0.89 x10^3^/µL, and platelet count of 28 x10^3^/µL. Lymph node trucut biopsy showed scattered singly and focal small collections of histiocytic cells in the background of lymphoplasmacytic cells. The histiocytes showed rounded or oval nuclei with abundant eosinophilic cytoplasm, mostly showing engulfed leukocytes. Occasionally lymphocytes were also seen. Small areas of necrosis surrounded by a histiocytic reaction in the form of poorly formed granulomas were also seen. Immunohistochemical workup showed CD68 positivity in histiocytes. Some cells (most likely immunoblasts) were positive for CD30 and negative for CD15.

The patient met five of eight HLH-2004 criteria necessary for diagnosis (including fever greater than 100.4°F for more than seven days, cytopenia, splenomegaly, elevated ferritin, elevated triglycerides, and hemophagocytosis in bone marrow) [4]. After ruling out all possible secondary causes of HLH, the patient was diagnosed as a case of primary HLH. Due to unavailability, genetic testing could not be performed.

**Figure 3:**
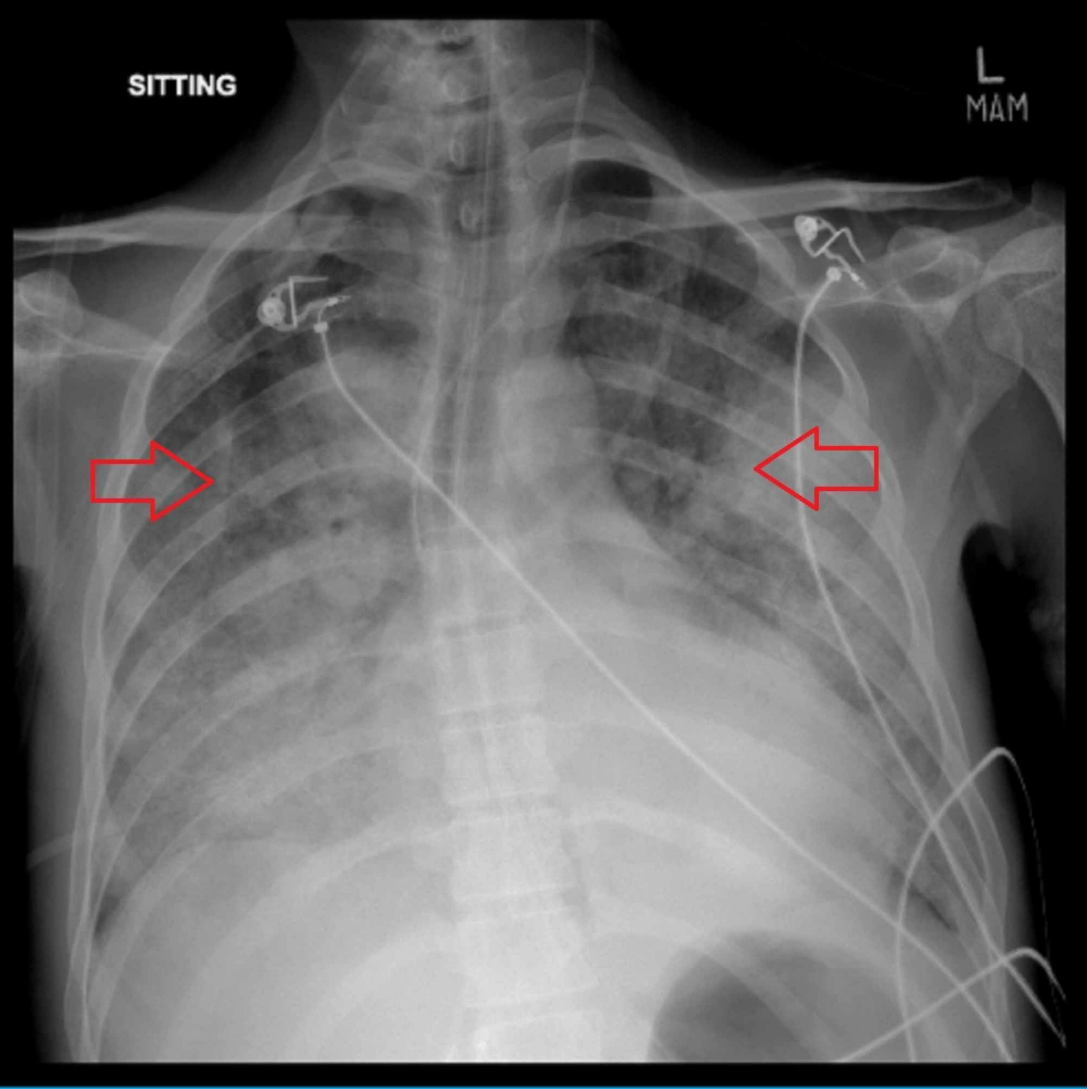
X-ray chest (AP view) showing worsening of pulmonary infilterates (red arrows). AP view: anterio-posterior view.

He was started on the HLH-2004 protocol (etoposide [VP16], cyclosporin-A [CSA], and dexamethasone [DEX]) along with empiric antituberculosis drugs (ATT) due to the presence of necrotic granulomas in the biopsy [4]. Unfortunately, on the second day of treatment patient’s condition deteriorated again. He developed severe metabolic acidosis, worsening of renal functions, and became hypotensive with increased lactic acidosis. The X-ray showed worsening of infiltrates (Figure 3), and he was again intubated. Antibiotics were further increased and inotropes were started. Despite adequate resuscitation measures, the patient did not improve. The family made the decision to withdraw life-support on the fourth day. The patient expired shortly after.

## Discussion

HLH was first described by Scott and Robb-Smith in 1939 as “histiocytic medullary reticulosis,” and a familial case of HLH was first reported by Farquhar and Claireaux in 1952 [5,6]. Literature is sparse on the incidence of HLH in adults, and the majority of the cases are seen in the pediatric population.

HLH has been mainly classified as primary and secondary. Under normal circumstances, natural killer (NK) cells and CD8+ cytotoxic T cells release perforins and granzymes (proteins that make pores and initiate apoptosis in target cells respectively). Perforin and granzymes are transported to the target cells, where they cause its destruction, thus eliminating the source of stimulus for immune activation. This removal of the stimulus leads to the down-regulation of the immune response of the body. In primary HLH, this normal phenomenon is hampered due to mutations in different genes. These mutations may produce a structural or functional defect in perforins or granzymes which cause decreased clearance of immune-stimulating antigens eventually leading to a persistently elevated immune response of the body [7]. Mutations causing primary HLH are summarized in Table 2 [3,7].

**Table 2:** Different mutations causing primary HLH [3,7]. A/R: autosomal recessive, X/R: X-linked recessive, HLH: hemophagocytic lymphohistiocytosis.

Primary HLH	Mode of inheritance	Genes involved
Familial hemophagocytic lymphohistiocytosis (FHL) (type 1-5)	A/R	PRF1, UNC13D, STX11, STXBP2
Chédiak–Higashi syndrome	A/R	LYST
Hermansky–Pudlak syndrome type 2	A/R	AP3B1
X‐linked lymphoproliferative disease (type 1 and 2)	X/R	SH2D1A, BIRC4
CD27 deficiency	A/R	CD27
Griscelli syndrome type 2	A/R	RAB27A
IL‐2‐inducible T‐cell kinase deficiency	A/R	ITK

Secondary or acquired HLH occurs due to infections, autoimmune disorders, malignancies, and drugs. Infectious causes are mainly viral (e.g. herpes simplex virus, EBV, CMV), but may include other parasitic and bacterial organisms such as Leishmania, Mycobacterium tuberculosis, or Salmonella typhimurium. Examples of autoimmune etiologies include systemic idiopathic juvenile arthritis, systemic lupus erythematosus (SLE), and macrophage activation syndrome (MAS). Malignancies often associated with acquired HLH include lymphoma and leukemias, such as NK/T‐cell lymphoma nasal type and anaplastic large cell lymphoma. HLH may be secondary to the use of drugs such as phenobarbital, carbamazepine, and sulfamethoxazole [3,7]. EBV has been identified as the most common infectious cause for both primary and secondary HLH [8]. The pattern of T-cell activation and differentiation in secondary HLH differs from primary HLH [9]. In this case, the patient presented with adult-onset primary HLH and no malignancy or infectious agent could be identified despite thorough investigations. The patient had no history of any drug use that could trigger HLH.

Clinically, HLH usually presents as a constellation of nonspecific signs and symptoms that make it a diagnostic challenge. Patients often present with fever, rash, hepatosplenomegaly, lymphadenopathy, arthralgia, neurologic dysfunction, and pancytopenia. Neurologic dysfunction in HLH is more commonly seen in a pediatric population. These symptoms often make physicians suspect a microbial infection or infestation. HLH should be thought of when these symptoms persist despite adequate antimicrobial treatments [10]. HLH can be misdiagnosed as a metabolic disorder, malignant lymphomas, and hemochromatosis [3]. In cases of severe HLH, patients may present with edema, shortness of breath, diarrhea, bleeding, and sepsis-like symptoms [10]. Henter et al. gave a revised criterion in 2004 to diagnose HLH [4]. Diagnosis of HLH can be made based on either molecular or clinical criteria. The criteria are summarized in Table 3 [4,7].

**Table 3:** HLH-2004 diagnostic criteria [4,7]. The diagnosis of HLH can be made if either clinical or molecular criteria is fulfilled. HLH: hemophagocytic lymphohistiocytosis.

Clinical criteria (any five out of eight necessary)	Molecular criteria
Fever	Mutations identified in PRF1, UNC13D, STX11, STXBP1, LYST, RAB27A, SH2D1A, BIRC4 or ITK genes
Splenomegaly	
Minimum of two cell lineages out of three affected in peripheral blood: hemoglobin (Hb) <9 g/dL (in <4-week infants: Hb<10 g/dL), platelets < 100×10^3^/µL, neutrophils < 1.0×10^3^/µL	
Hypertriglyceridemia and/or hypofibrinogenemia: fasting triglycerides ≥265 mg/dL (3.0 mmol/L), fibrinogen ≤150 mg/dL	
Hemophagocytosis in bone marrow, spleen, or lymph nodes and no evidence of malignancy	
Reduced or absent natural killer cell activity	
Ferritin ≥500 ng/mL	
Elevated soluble IL-2 receptor (soluble CD25) ≥2,400 U/mL	

In our case, the patient met at least five out of eight conditions necessary for the diagnosis of HLH. He presented with fever, hepatosplenomegaly, high serum levels of triglycerides and ferritin, hemophagocytosis in bone marrow, and a decreasing cell count.

With a wide range of symptoms, many differentials can be considered in the case of HLH. They include MAS, any infection by the above-mentioned organisms, liver failure, multiple organ dysfunction syndromes, encephalitis, autoimmune disorders, drug reaction with eosinophilia and systemic symptoms, child abuse, transfusion-associated graft-versus-host disease, and cytophagic histiocytic panniculitis [11]. In this case, a differential of infectious causes (mainly tuberculosis and EBV), malignancy (lymphoma/ leukemia), and autoimmune causes (mainly SLE) were made. All these were ruled out through proper investigations.

To make a confirmatory diagnosis of HLH, a series of investigations are needed. Initial investigations include complete blood count, liver function tests (LFTs; alanine aminotransferase, aspartate aminotransferase, gamma-glutamyl transferase, and bilirubin), renal function tests (blood urea nitrogen and creatinine), coagulation profile (prothrombin time, partial thromboplastin time, fibrinogen, and D-dimer), iron profile (including serum ferritin), and serum triglyceride level. Further investigations include blood, urine, and cerebrospinal fluid (CSF) cultures, viral count, CT scan, ultrasound (US) abdomen, CD-25 count, autoimmune workup, genetic analysis, lymph node, and bone marrow biopsy [12]. CBC shows a reduced number of cells, and LFTs show abnormal levels of bilirubin and liver enzymes. Lymphadenopathy and organomegaly can be noted on the US and CT scan. In primary HLH, genetic analysis will show a mutation in genes (mentioned above) responsible for regulating NK and T-cell functions. In cases of secondary HLH, viral markers, blood cultures, or autoimmune workup will be positive. In this case, CSF culture was not performed as there were no signs of meningitis or encephalitis. All investigations for secondary HLH were found negative. Genetic analysis could not be performed due to a lack of facility. A final diagnosis of primary HLH was made after excluding all secondary causes.

Corticosteroids are considered first-line treatment for HLH except in cases of lymphoma-associated HLH. The goal of initial treatment is to lower the hyperactivity of the immune system. Along with corticosteroids, prophylactic antimicrobials (trimethoprim‐sulfamethoxazole + antimycotic agent) should be started and supportive treatment for the impaired hepatic and renal function should be provided [7]. HLH-94 (VP-16 and DEX) and HLH-2004 (CSA, VP-16, and DEX) protocols are widely used for the treatment of HLH [2,4,7,12]. Allogeneic hematopoietic stem cell transplantation (allo-HSCT) should be considered in patients showing response to immunochemotherapy with CSA, VP-16, and DEX. Three-year survival probability in patients undergoing HSCT is 64% [4].

EBV-associated HLH can be treated with the same protocol as primary HLH. Other infection-associated HLH can be treated with corticosteroids, CSA, and intravenous immunoglobulin along with specific treatment for that infection. HLH secondary to lymphoma can be treated with CSA and corticosteroids in addition to chemotherapy targeted against specific malignancy. Intrathecal injections of hydrocortisone and methotrexate are indicated in the central nervous system (CNS) involvement [4]. CNS involvement reduces the survival rate in HLH patients compared to those who do not have such lesions (40% vs 67%) [13]. Therefore, allo-HSCT should be done before the involvement of CNS [7]. In this case, empiric antibiotics were started before the diagnosis was reached. Upon confirmation of diagnosis, the HLH-2004 protocol was followed along with empiric ATT therapy, but the patient did not respond to treatment.

HLH is a fatal disease and the prognosis is very poor. Children have a five-year survival probability of 54% with worse outcomes in infants less than six months of age [2,3]. Retrospective data show that adults with HLH had a median survival of four months. The survival period was even lower in patients with malignancy-associated HLH (median 2.8 months). Allo-HSCT patients had a longer median survival period of 21.5 months [14].

## Conclusions

HLH is a very rare and life-threatening immune disorder. It requires skilled and timely management. Due to its similarity with various infections and malignancies, differentiating the disorder can be puzzling. Physicians should promptly investigate the patient who is not responding to antimicrobial therapies for other causes. Diagnosing primary HLH in a resource-limited setting can be a challenge as genetic testing is not available. All possible causes of secondary HLH should be eliminated before treating the patient as a case of primary HLH.
